# Jasmonate, salicylate, and ethylene-responsive transcriptomics discovery in spikelets of three wheat genotypes reveals a rapid and conserved response for jasmonate signaling

**DOI:** 10.1080/15592324.2026.2679322

**Published:** 2026-06-26

**Authors:** Nora A. Foroud, Ravinder K. Goyal, Ziying Liu, Yifeng Li, Dianevys González-Peña Fundora, Anas Eranthodi, Daria Ryabova, André Laroche, Michele C. Loewen, Youlian Pan

**Affiliations:** a Lethbridge Research and Development Centre, Agriculture and Agri-Food Canada, Lethbridge, Alberta, Canada; b Digital Technologies Research Centre, National Research Council of Canada, Ottawa, Ontario, Canada; c Department of Computer Science, Brock University, St. Catharines, Ontario, Canada; d Industrial Research Assistance Program, National Research Council of Canada, Ottawa, Ontario, Canada

**Keywords:** Ethylene, inflorescence, jasmonic acid, wheat, plant hormones, RNA-sequencing, salicylic acid

## Abstract

The accumulation of plant hormones in different tissues leads to transcriptional reprogramming that guides the downstream phenotypic response. Current models are largely derived from *Arabidopsis*, and increasing evidence indicates that monocots only partially conform to these canonical pathways, with additional variability even within the clade. In cereals, salicylate (SA), jasmonate (JA), and ethylene (ET) signaling play important roles in both inflorescence development and pathogen defense. This communication focuses on the transcriptional responses of wheat inflorescence tissue in three genotypes 8 h following exogenous applications of salicylic acid (SA), methyl jasmonate (MeJA), and the ethylene-releasing compound, ethephon (ETp). Nearly 8000 differentially expressed genes (DEGs) were detected in response to MeJA, approximately half of which were conserved across all three genotypes. In contrast, SA and ETp elicited limited responses, each inducing fewer than 100 DEGs with little to no overlap among genotypes. The comparatively feeble response to SA and ETp suggests a delayed response compared to MeJA. Other studies have assessed differential transcriptomes at 24 h or later. Notably, other studies in cereals, including wheat, have also reported less pronounced transcriptional changes to salicylate and ethylene signaling. These findings demonstrate both conservation and genotype-specific variation in the transcriptional response to plant hormones.

## Introduction

Plant hormones are signaling molecules involved in coordinating various developmental processes and defense responses through the regulation of gene expression. Salicylate (salicylic acid; SA), jasmonate (jasmonic acid; JA), and ethylene (ET) signaling pathways are the classic plant defense hormones^1^ that are also involved in flowering and inflorescence development.^2^ Plant hormone pathways are complex, involving both synergistic and antagonistic cross-talk among various members.^3^

SA signaling plays a role in flowering time and pollen tip growth in *Arabidopsis*,^4^ ^,^ ^5^ but it is most widely known for its ability to induce systemic acquired resistance to plant disease and activation of the hypersensitive response and localized bursts of programmed cell death.^6^ ^,^ ^7^ This type of response can be quite effective in preventing the growth or spread of obligate biotrophs, but is generally less effective against most necrotrophic pathogens. On the other hand, JA and ET frequently act synergistically to overcome the actions of necrotrophic pathogens.^1^ JA is a linolenic acid- derived oxylipin signaling molecule^8^ involved in flowering, stamen development, and pollen maturation.^9-11^ On the plant defense side, JA is activated by various insects and pathogens^12-14^ and involves expression of defense-related genes, such as *Plant defensins* (*PDFs*) and *systeminS*, and genes involved in lignin biosynthesis.^11^ ^,^ ^15^ ^,^ ^16^ JA is also a positive regulator of the ET biosynthesis pathway.^17-19^ ET is known for its role in senescence and fruit ripening.^20^ ^,^ ^21^ While senescence may be advantageous to some pathogens,^22^ coordinated activity of ET with JA signaling can also provide resistance against some plant pathogens.^23^ ^,^ ^24^ Albeit less common, antagonistic interactions have also been reported between ET and JA.^25^ ^,^ ^26^ By contrast, while positive interactions exist between SA and JA, antagonism is more common in this case.^1^ ^,^ ^27-30^ On the other hand, it has been reported that ET can block SA-mediated suppression of JA signaling.^31^ ^,^ ^32^ The interplay among hormone signaling pathways represents a delicate balancing act of antagonistic and synergistic interactions, constituting an adaptive strategy in the plant defense arsenal to fine-tune and focus the plant’s response to a particular threat, often resulting in a compromise between fitness and survival.^33-35^

Our knowledge of hormone-responsive genes is heavily biased towards *Arabidopsis* and other dicotyledonous plants; however, these pathways may differ between dicots and monocots. In fact, a smattering of evidence accumulated over the years indicates that there are important differences in the SA signaling pathway amongst these two clades.^36-40^ Taking it one step further, there appear to be major differences in the SA and ET response within the monocot clade, as reported by Kakei et al. in their observation of hormone-responsive transcriptomes in *Brachypodium distachyon*, a model crop for cereal species.^41^ Since these classic plant defense hormones (SA, JA and ET) are also involved flower/inflorescence development and ripening, they are especially interesting in the context of the wheat spike which is susceptible to fungal diseases, such as common bunt and Fusarium head blight.^42^ ^,^ ^43^

Wheat is one of the most important staple crops, with roughly 700 million tonnes produced annually around the world.^44^ As an important step towards better understanding of hormone signaling in wheat, Qi et al. published a reference map for different hormone transcriptional responses of the wheat spike in “Roblin”.^45^ Heckmann et al. furthered these efforts in their evaluation of the JA and SA transcriptomic response in the leaves of “Remus” and “CM82036”, where a set of wheat marker genes were reported for these two pathways.^46^ In this study, transcriptional profiling was carried out on the inflorescence of three different wheat genotypes, following exogenous applications of SA, methyl-JA (MeJA), and ethephon (ETp; a chemical precursor to ethylene). The results highlight species-specific differences in the host response to plant hormones, while also demonstrating that genotype-specific differences can occur even within individual species.

## Materials and methods

### Plant material and chemical treatments

Three wheat genotypes were used in this experiment: Canadian Hard Red Spring cultivar “Superb”,^47^ and two related doubled haploid lines, GS-1-EM0040 (“CIMMYT11”/“Superb”*2) and GS-1-EM0168 (“CM82036”/“Superb”*2).^48^ The doubled haploid lines GS-1-EM0040 and GS-1-EM0168 will henceforth be respectively referred to as DH1 and DH2. Plants were grown in a greenhouse at 22/18°C (day/night) with a 16 h photoperiod as previously described.^48^ When the plants reached anthesis, the spikes were sprayed to run-off with: (i) water; (ii) SA (Sigma S-0875), (iii) MeJA (Sigma 392707), or (iv) 2-chloroethylphosphonic acid (ETp) (Sigma C0143). The hormone solutions were prepared fresh (within 20 min of application) to a final concentration of 100  µM in water; the concentration was selected based on previous successes with these treatments in wheat.^36^ ^,^ ^49^ ^,^ ^50^ The treatment groups were held in separate chambers in order to prevent cross-talk among the signaling pathways. Spikelets collected from five plants within each treatment group were harvested 8 h after treatment, frozen in liquid nitrogen, and stored at -80°C until used for RNA isolation. The 8 h harvest time was selected based on previous work with these three wheat genotypes, where a microarray study revealed transcriptional changes in spikelets adjacent to *Fusarium* *graminearum-*inoculated spikelets at 8 h after treatment.^48^ Each experiment was run in triplicate.

### RNA isolation and sequencing

For each of three replicates, spikelets collected from each of five spikes 8 h after chemical or water treatments were ground to a powder in liquid nitrogen with a mortar and pestle. RNA was isolated from 1 g of powdered spikelets with TRIzol^TM^ Reagent (Invitrogen^TM^) according to the manufacturer’s instructions. The pellet was resuspended in 50  µL of RNase-free Optima^TM^ water (Fisher Scientific) and transferred to an RNeasy MiniElute Cleanup kit. DNase digestion was carried out with RNase-Free DNase I (Thermo ScientificTM) as previously described.^51^ RNA quality was assessed with a 2100 Bioanalyzer (Agilent Genomics). RNA library preparations and HiSeq were carried out through the NRC Sequencing Facility in Saskatoon, SK, as previously described.^52^ The raw sequence reads are available at the NCBI GEO repository (GSE161955).

### RNA-seq data analysis

RNA-seq reads were pre-processed by trimming adapter and barcode sequences, filtering out low-quality (Phred Score ≤ 20) (http://www.phrap.com/phred) and short reads (length ≤ 20 bps).^53^ An average of 16 million paired reads per sample remained for mapping against the high-confidence gene models from the IWGSC RefSeq Version 2.1 wheat reference genome.^54^ The cleaned RNA-seq reads from each sample were mapped against the reference genome using STAR v2.7.10a^55^ to generate gene-level counts. The RSEM software package^56^ was then used to quantify mRNA levels in each sample, expressed as transcripts per million (TPM), from BAM files generated in STAR. Data normalization and differentially expressed gene (DEG) analyses for each pairwise comparison were carried out in DESeq2.^57^ A criterion of log2-fold change (FC) ≥ 1, *p* ≤ 0.05 was applied for DEGs. Within each genotype, a DEG was obtained by comparing hormone-treated wheat spikes to those treated with water. In addition, a minimum expression threshold of 2 TPM was applied, such that the higher expressed sample in each pair had at least 2 TPM.

Differences and similarities were compared among the hormone treatments within a genotype, and for the same hormone treatment across the three genotypes. The data were then partitioned into different groups, according to the Differential Expression Feature Extraction (DEFE) method described in Pan et al.^58^ Two sets of groupings were generated, one for hormone treatments and the other for genotypes. Each group was assigned a pattern ID starting with an “H” or “G”, followed by three digits, each representing a wheat genotype or a hormone treatment within a genotype; “0” denotes no change, “1” for up- and “2” for down-regulation. The prefix “H” denotes the hormonal response profile within a genotype, whereas “G” denotes genotypic variation in response to specific hormone treatments. For the “H” series, the numbers in the first, second, and third positions represent SA, MeJA, and ETp, respectively. For example, H_DH1_012 represents a group of genes in DH1 for which no change was observed in response to SA treatment, but up-regulation occurred in response to MeJA and down-regulation in response to ETp. Likewise, the similarities and differences in the response to a specific hormone treatment *versus* the water control among the three wheat genotypes are represented by the “G” series DEFE patterns; these comparisons across genotypes are labeled as G (DH1, DH2, and Superb). For example, G_SA_111 denotes a group of genes that are up-regulated by SA treatment in all three genotypes.

The WGCNA R package^59^ was used to cluster the DEGs and to perform gene-associated network analyses. The Pearson correlation coefficient matrix *C* was first computed, and split into *C*_*+*_ and *C*_*-*_ for positive and negative correlations, followed by positive and then negative adjacency matrices, *A*_*+*_ and *A*_*-*_, according to the following calculation: $A={\mathrm{|C|}}^{p}$ , where *p* is the power for soft-thresholding. A topology overlap similarity matrix (TOM) was generated for each of the two adjacency matrices, and the two TOM matrices were subsequently merged into a similarity matrix using the formula:

(1) $$S=\frac{\mathrm{Tomsimilarity}\left(A_{+}\right)+\mathrm{Tomsimilarity}(A_{-})}{2}$$

The similarity matrix (1-*S* as distance measure) was used for hierarchical clustering, and the Dynamic Tree Cut method^59^ was employed to generate gene clusters/modules. Gene-trait and cluster-trait correlation matrices were computed in WGCNA. Gene association network analysis was performed by using the similarity matrix (***S*** in Equation (1)) as edge weights. The top 1% weight in ***S*** was considered a valid edge in the network. Highly connected genes (>1000 connections) were considered key hub genes.

Gene orthologues were obtained from *B. distachyon* (JGI: https://jgi.doe.gov/), *Oryza sativa* (https://plants.ensembl.org/Oryza_sativa/Info/Index), and *A. thaliana* (TAIR: www.arabidopsis.org). Gene Ontology (GO)-gene associations were obtained from Ensembl Plants release 51 and IWGSC-Refseqv2.1 as described in Pan et al.^60^ GO enrichment analysis was carried out using an updated version (https://github.com/DTNRC/GOAL2.0) of the Gene Ontology AnaLyzer (*GOAL*) software.^61^ To further enhance and verify the gene descriptions, the predicted protein sequences were analyzed for protein domains using ExPASy PROSITE freeware (https://prosite.expasy.org/). Principal component analysis (PCA) was performed using the PCAtools R package in Bioconductor^62^ to assess variation and relatedness within the RNA-Seq datasets. Heatmaps were prepared for the transcripts from each dataset using the ComplexHeatmap R package in Bioconductor.^63^

## Results and discussion

**Figure 1.**
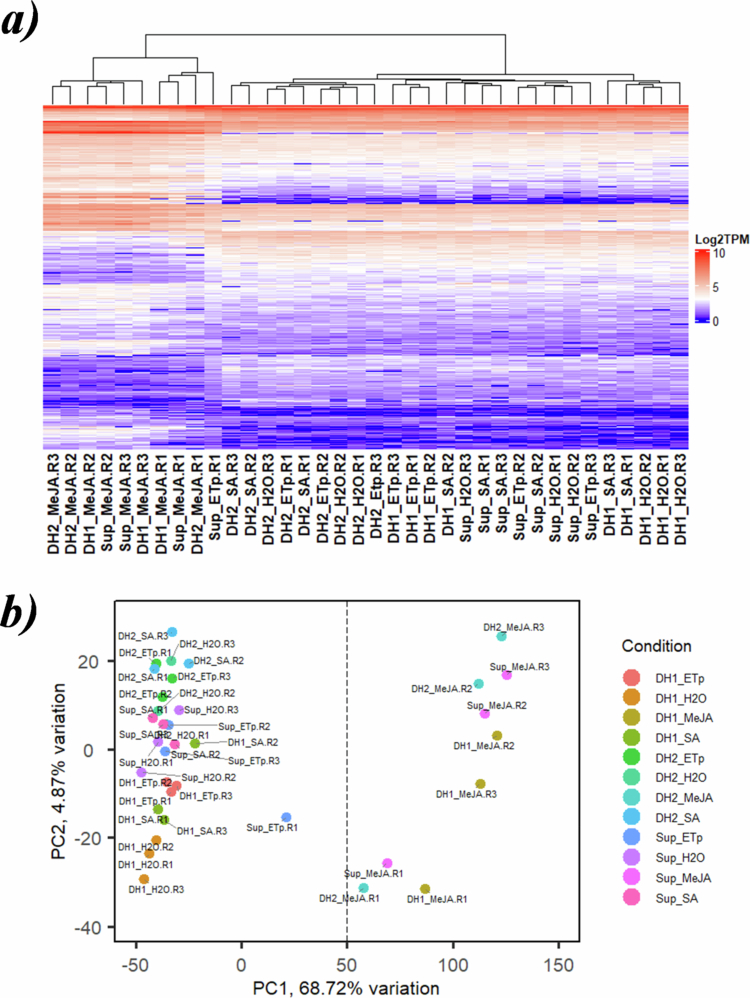
Transcriptome overview. (*a*) Heatmap, (*b*) principal component analysis.

**Figure 2.**
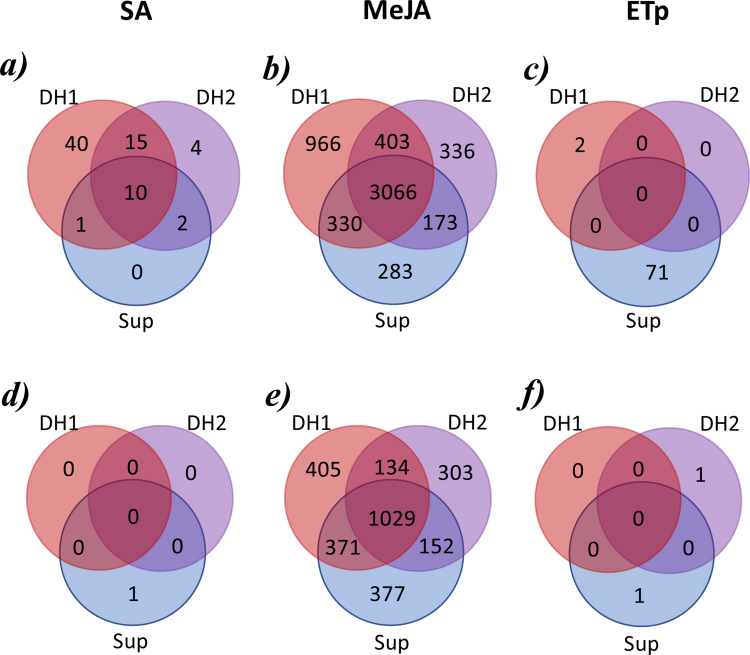
Venn diagram showing hormone-responsive DEGs common and specific to each individual among the three wheat genotypes. Top row (*a*, *b*, *c*): up-regulated, joint of three hormone treatment (% SA, MeJA, ETp) = 14, 55, 0. Bottom row (*d*, *e*, *f*): down-regulated, joint of three hormone treatment (% SA, MeJA, ETp) = 0, 37, 0.

On average, 96% of the 16 million paired-end RNA-seq reads per sample were mapped to the wheat reference genome. A total of 8369 DEGs (log2 FC ≥ 1, *p* ≤ 0.05) were identified and are listed in Supplementary File 1. A global view of the DEG dataset obtained by clustering samples based on the DEGs indicated that the profiles in response to the MeJA treatment revealed coherence among the three genotypes (Supplementary Figure 1). By contrast, ETp and SA treatments were clustered within the respective genotypes. Similar observations were made in the heatmap and principal component analysis (PCA) (Figure 1). These results are consistent with the fact that the MeJA treatment resulted in a substantially higher number, by two orders of magnitude, of DEGs than the ETp or SA treatments (Supplementary Table 1); the three genotypes shared 49% of the DEGs as a result of MeJA treatment (Figure 2). Unanimously across all three genotypes, 3066 genes (DEFE pattern G_MeJA_111) were up-regulated and 1029 (G_MeJA_222) down-regulated as a result of MeJA treatment (Supplementary Table 2). Collectively in any of the three genotypes, 72 and 73 genes were up-regulated, and 1 and 2 down-regulated as a result of SA and ETp treatments, respectively (Figure 2, Supplementary Table 2). In response to SA treatments, unanimously across all three genotypes, 10 genes were up-regulated (G_SA_111), whereas no common DEGs were observed across any two genotypes as a result of ETp treatment.

DH1 exhibited the strongest overall transcriptional response, with 6,750 DEGs across all hormone treatments (Supplementary Table 3). MeJA uniquely regulated 966 up-regulated and 405 down-regulated genes, whereas SA and ETp had minimal effects (SA: 40 up, 0 down; ETp: 2 up, 0 down; patterns G100 and G200, Supplementary Table 2; Figure 2). A similar pattern was observed in DH2 and “Superb”, where MeJA accounted for the majority of uniquely regulated genes (Figure 2). Consistent with this, thousands of genes were uniquely responsive to MeJA within each genotype compared with the other treatments (patterns H010 and H020; Supplementary Table 3). Notably, the stronger response in DH1 mirrors previous transcriptome analyses of these same genotypes following treatments with either *F. graminearum* or the mycotoxin deoxynivalenol, where DH1 also exhibited the strongest transcriptional response compared with the other two genotypes.^48^

Seventeen DEG clusters were observed, 13 of which were significantly (*p* ≤ 0.05) correlated with MeJA treatment (7 positively and 6 negatively), one was positively correlated with SA, and none with ETp (Supplementary Table 4). Similarly, Pearson correlation analysis showed that more than an order of magnitude higher number of genes was significantly (*p* ≤ 0.05) correlated with MeJA (6806) than with SA (275) or ETp (187). All but one of the MeJA-correlated genes were differentially expressed in response to the MeJA treatment in at least one genotype, while 55% were differentially expressed across all three genotypes (G_MeJA_111 and G_MeJA_222, Supplementary Table 2). A total of 206 DEGs were highly connected (connection degree ≥ 1,000; see red bold text in column DN of Supplementary File 1) in the gene association networks, and all were up-regulated by MeJA across the three genotypes.

The MeJA treatments resulted in up-regulation of the expected JA response pathways, as reflected in the GO analysis (Supplementary Table 5). Among the up-regulated genes, many are involved in JA signaling and biosynthesis; for example, 8 encode 12-oxophytodienoate reductase (OPR), and 7 encode 12-oxophytodienoate reductase-like protein (Supplementary Table 6). The MeJA responsive genes also encompassed GO terms associated with various defense response machineries (Supplementary Table 7), including some of the expected secondary metabolism pathways known to involve JA signaling (for example, “phenylpropanoid metabolic process” (*p* < 10^−10^, GO:0009698), “cinnamic acid metabolic process” (*p* < 10^−5^, GO:0009803), and “lignin biosynthetic process” (*p* < 10^−5^; GO:0009809). In contrast, the 1029 genes unanimously down-regulated by MeJA were enriched in photosynthesis and other primary metabolism-related GOs, such as “photosynthesis, light reaction” (*p* < 10^−40^, GO:0019684; Supplementary Table 8).

Each of the 10 genes universally up-regulated as a result of SA treatment (G_SA_111 in Supplementary Table 9) was also significantly (*p* < 10^−5^) correlated with SA treatment, in the clusters significantly correlated (*p* < 10^−6^) with SA treatment. By contrast, in the ETp treatment, there were no commonly regulated DEGs among the three wheat genotypes. Of the 73 DEGs up-regulated by ETp treatments, 71 (97%) were uniquely up-regulated in Superb, two in DH1 (Figure 2c). Qi et al. similarly reported a subdued response to SA in the spikelets of wheat cv. “Roblin” compared with MeJA treatments,^45^ and Kakei et al. found that no ET-responsive genes were detected in *Brachypodium* unless they analyzed the dataset at a low stringency.^41^ It was surprising to find so few commonalities across the three wheat genotypes by SA or ETp treatments. The possibility that basal levels of SA or ET differ among the three wheat genotypes evaluated here cannot be discounted, in which case some of the SA-upregulated genes in one genotype, for example, could already be elevated in other genotypes.

Among the 10 unanimously up-regulated SA-responsive genes, two (TraesCS1B03G0695700, TraesCS1D03G0572300) are involved in the regulation of systemic acquired resistance, one (TraesCS2B03G1049900) encodes salicylic acid 3-hydroxylase (EC:1.14.11.-), and three (TraesCS5B03G0589800, TraesCS5B03G0589900, and TraesCS5D03G0541500) encode the WRKY transcription factor WRKY76-like (Supplementary Table 9). Interestingly, up-regulation of *TaWRKY45-B* (TraesCS2B03G1299500), reported as a wheat-specific marker identified in leaf tissue,^46^ was the only up-regulated gene in “Superb” spikelets following the SA treatments in the current study. Two other *WRKY45* genes, TraesCS2A03G1143500 and TraesCS2D03G1093400, which are orthologous to the *B. distachyon* SA markers,^41^ were also up-regulated by SA in the present study, but again only in “Superb”.

Unfortunately, in the case of the ETp treatments, there was no consensus among the DEGs across the three wheat genotypes. There may also be signaling activities beyond altered gene expression in place at 8 h post-treatment. For example, various proteins may already be in place waiting to come into action. Several enzymes involved in the ET biosynthesis and response pathways are post-translationally regulated by reversible phosphorylation.^64-66^ Interestingly, in *Zoysia japonica* leaves, ETp treatment resulted in a reduced activity of key ET biosynthesis enzymes, such as 1-aminocyclopropane-1-carboxylic (ACC) synthase (ACS) and ACC oxidase (ACO), and reduced the accumulation of the ET precursor ACC.^67^ Thus, a negative feedback loop for ET biosynthesis may occur in the presence of ET released from ETp treatments. It may be a worthwhile pursuit to determine whether changes in enzymatic activity, or activation of transcription factors, are more prominent than actual changes in gene expression in ETp treatment of wheat spikes at 8 h post-treatment. The total number of DEGs observed in the ETp treatment in any of the wheat genotypes was relatively small (75 total), especially when compared to that of the MeJA treatments. In fact, the same is true for SA treatments, which did lead to a stronger response than ETp, but were nonetheless relatively small in scale. This points to the possibility that the 8 h harvest time did not capture the most significant transcriptional changes in response to 100  µM of ETp or SA spray treatments in wheat spikes.

While a conserved transcriptional response of JA has been reported across different species from monocots to dicots, it is interesting that the same is not true for SA or ET.^41^ In fact, differences in both SA and ET signaling have been reported between two monocot model crops: rice and *Brachypodium*.^41^ Furthermore, a comparison of results from different reports already suggests that the SA response may not be conserved even between different wheat genotypes. In *Arabidopsis*, *pathogenesis-related 1* (*PR1*) is a well-known marker of SA signaling,^68^ and *PR1* was also found to be SA-regulated in *B. distachyon*.^41^ However, cumulative evidence demonstrates that *PR1* and other pathogenesis-related (PR) genes may not be suitable markers to differentiate SA *vs* JA responses, as recently stated by Heckmann et al.^46^ who systematically tracked the expression of *TaPR1*, *TaPR2,* and *TaPR5* gene families in wheat leaves treated with SA and MeJA. Earlier, it was shown that neither *TaPR1.1* nor *TaPR1.2* was induced in the leaves of cv. Kanzler at one or 4 d after 3  mM applications of SA, or 300  µM of benzothiadiazole,^38^ a synthetic analog of SA. On the other hand, *TaPR1.2*, but not *TaPR1.1*, was up-regulated in the seedlings of cv. Laura at 2 and 3 d after the exogenous applications of 100  µM SA.^36^ Interestingly, both genes were up-regulated by 100  µM MeJA treatments.^36^ Of course, these differences may be attributed to various aspects of the experimental design used in these two studies, including the concentration of applied SA, the developmental stage investigated, the genotypes used, and/or the incubation time after treatment. In the present study, a number of the *TaPR1* genes identified by Heckmann et al.^46^ were differentially regulated, but only in response to the MeJA treatments, as were *TaPR2*, *TaPR3,* and *TaPR5*, with genotypic differences for some genes within these families (Supplementary Table 10).

Different findings on *PR* gene expression in wheat among studies support the idea that different wheat genotypes vary in their response to hormone signals. Such variation can be an indicator of genotype-specific response to different hormone-related traits, such as disease resistance. Makandar et al.^69^ found that two cultivars that differ in Fusarium head blight resistance responded differently to benzothiadiazole application: in the resistant cultivar, “Sumai 3”, *PR1* expression was observed as early as 6 h after a treatment with 100  µM benzothiadiazole, whereas the same concentration or more had no effect at 6 h in the susceptible cultivar, “Bobwhite”. Meanwhile, “Bobwhite” showed up-regulation of this gene by 12 h after a higher dosage of benzothiadiazole (200 µM), and at 24 h in response to the lower dose of 100 µM. Thus, the differences in hormone signaling among genotypes may contribute to resistance or susceptibility to biotic stresses, and some of these differences may be temporal or related to different levels of sensitivity to different hormone concentrations. Bearing that in mind, the RNA-seq data presented herein provide a snapshot of differential gene expression at 8 h after treatment, using a single concentration of each elicitor. Therefore, it is possible that the genotypic difference observed here is a difference in the timing and/or sensitivity of the response. For example, one genotype may respond more rapidly to SA or ETp treatments than another, as already reported for SA treatments in “Bobwhite” vs. “Sumai 3”.^69^ The difference in timing and/or sensitivity of response to hormone treatments in the three genotypes in the current work may provide insight into how these genotypes differ in their response to diseases of the wheat inflorescence, such as Fusarium head blight.^48^

Whether the genotype-specific behavior observed in the SA and ETp treatments reflects differences in temporal dynamics or more fundamental differences in gene regulation among wheat genotypes, this study provides the first evidence of genotype-specific variation in global SA and ETp transcriptomic responses in wheat. This concept is not entirely foreign; van Leeuwan et al.^70^ observed SA-responsive DEGs in a microarray study comparing seven *A. thaliana* accessions and found that, while similarities were observed in the gene networks affected, there was nonetheless significant variation in DEGs among accessions.^70^ The authors proposed that these genotype-specific variations may explain different phenotypes among different accessions, for example, to specific plant diseases.

Interestingly, an analysis of the orthologues to SA, JA, and ET marker genes identified from *B. distachyon* leaves^41^ and of SA and JA marker genes from wheat leaves^46^ shows a conserved JA response, but deviates in the SA and ET responses (Supplementary Table 11). All of the proposed JA marker genes were up-regulated in each of the three wheat genotypes (with the exception of JAMyb-4A) in response to the MeJA treatment. On the other hand, only a subset of the SA markers, specifically those reported for wheat leaves,^46^ were differentially regulated in wheat spikelets and only in the DH1 genotype, thus showing a genotype-specific response. Meanwhile, none of the ET markers^41^ were differentially regulated by the ETp treatment, although the exceptionally low number of DEGs identified in the ETp treatment may explain these results.

There are numerous examples in the scientific literature where altered expression of specific genes is used to infer the role of specific plant hormones in the trait of interest.^48^ ^,^ ^71-73^ In some cases, these are part of larger studies where the role of hormones is not emphasized, and in others, the key message of the work is to relay the importance of a specific hormone pathway in regulating that trait, often accompanied by appropriate validation. Cumulative evidence clearly indicates that hormone-regulated gene networks in monocots diverge from the established transcriptional markers of these pathways in dicots, with additional variation observed among monocot species. By investigating early transcriptional changes in response to SA, MeJA, and ETp treatments, the present study explored the variation of hormone signaling responses in the spikelets of different wheat genotypes. The results showed that while signaling from the JA pathway was strong and fairly conserved, SA and ET signaling responses were less pronounced and varied among the three genotypes assessed. A follow-up study to include identification of the temporal maximum response and the effect of treatment concentrations would provide further insights into how and where these individual pathways are conserved, and point to how and where they are divergent, among the different wheat genotypes. The results presented herein, along with other examples in the literature,^41^ ^,^ ^69^ ^,^ ^70^ emphasize the importance of validating the role of a specific hormone in a given trait and confirming that putative marker genes are in fact regulated by a specific hormone in a given genotype.

## Supplementary Material

Foroudetal_Hormone_RNASeq_20260515_SuppTables.xlsx Foroudetal_Hormone_RNASeq_20260515_SuppTables.xlsx

Foroudetal_Hormone_RNASeq_20260515_SuppFile1.xlsx Foroudetal_Hormone_RNASeq_20260515_SuppFile1.xlsx

Foroudetal_Hormone_RNASeq_20260416_SuppFig1.docx Foroudetal_Hormone_RNASeq_20260416_SuppFig1.docx

## Data Availability

The raw sequence reads are available at the NCBI GEO repository (GSE161955).
