# Supplementary figures and images for: Jasmonate, salicylate, and ethylene-responsive transcriptomics discovery in spikelets of three wheat genotypes reveals a rapid and conserved response for jasmonate signaling
Source: Plant Signal Behav. 2026 Jun 26;21(1):2679322. doi: 10.1080/15592324.2026.2679322 (PMC13313262; doi:10.1080/15592324.2026.2679322)

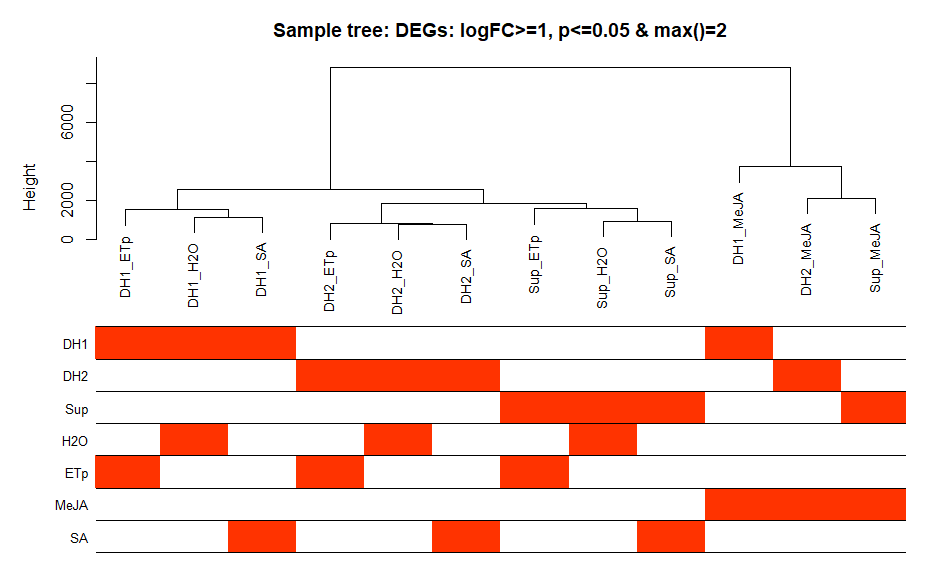


**Supplementary File 1.** The hierarchical relationship among the samples.

Supplement: Foroudetal_Hormone_RNASeq_20260416_SuppFig1.docx [file KPSB_A_2679322_SM4193.docx]
